# Whole fungal elicitors boost paclitaxel biosynthesis induction in *Corylus avellana* cell culture

**DOI:** 10.1371/journal.pone.0236191

**Published:** 2020-07-16

**Authors:** Mina Salehi, Ahmad Moieni, Naser Safaie, Siamak Farhadi

**Affiliations:** 1 Department of Plant Breeding and Biotechnology, Faculty of Agriculture, Tarbiat Modares University, Tehran, Iran; 2 Department of Plant Pathology, Faculty of Agriculture, Tarbiat Modares University, Tehran, Iran; Universite d'Orleans, FRANCE

## Abstract

Paclitaxel is an effective natural-source chemotherapeutic agent commonly applied to treat a vast range of cancers. *In vitro Corylus avellana* culture has been reported as a promising and inexpensive system for paclitaxel production. Fungal elicitors have been made known as the most efficient strategy for the biosynthesis induction of secondary metabolites in plant *in vitro* culture. In this research, *C*. *avellana* cell suspension culture (CSC) was exposed to cell extract (CE) and culture filtrate (CF) derived from *Camarosporomyces flavigenus*, either individually or combined treatment, in mid and late log phase. There is no report on the use of whole fungal elicitors (the combined treatment of CE and CF) for the elicitation of secondary metabolite biosynthesis in plant *in vitro* culture. The combined treatment of CE and CF significantly led to more paclitaxel biosynthesis and secretion than the individual use of them. Also, multivariate statistical approaches including stepwise regression (SR), ordinary least squares regression (OLSR), principal component regression (PCR) and partial least squares regression (PLSR) were used to model and predict paclitaxel biosynthesis and secretion. Based on value account for (VAF), root mean square error (RMSE), coefficient of determination (R^2^), mean absolute percentage error (MAPE) and relative percent difference (RPD) can be concluded that mentioned regression models effectively worked only for modeling and predicting extracellular paclitaxel portion in *C*. *avellana* cell culture.

## Introduction

Paclitaxel is a well-known chemotherapeutic agent widely applied as a therapy for various types of cancers [1], and this is also used as a treatment for non-cancerous human diseases [2]. Paclitaxel as a cytoskeletal drug arrests the proliferation of tumor cells following stabilizing the microtubules. Indeed, this valuable metabolite blocks cell cycle in G0/G1 and G2/M phases by interaction with tubulin [3].

This fantastic diterpene alkaloid, paclitaxel, was originally found in the bark of *Taxus brevifolia* tree [4]. Since *Taxus* trees are slow-growing and harvesting the bark is destructive [5], thus the continuous harvest of native plant bark for commercial production of paclitaxel was untenable. Nowadays, plant cell factories offer a promising and environment-friendly approach for large-scale paclitaxel production [6–10]. The rising demand for paclitaxel, and *Taxus* recalcitrant behavior under *in vitro* conditions have caused extensive efforts toward finding alternatives for producing this invaluable secondary metabolite.

*In vitro* culture of *Corylus avellana* (European filbert) has been made known as a promising and inexpensive strategy for producing paclitaxel [7–12]. The major advantage of producing paclitaxel through hazel cell culture is that *in vitro* culture of *C*. *avellana* is easier as compared to that of *Taxus*, and given that *C*. *avellana* is a dicotyledonous plant, possibly *C*. *avellana* response to genetic manipulation by *Agrobacterium* is more promising than that of *Taxus* [7–10]. Obtaining high-producing *in vitro* cultures is a key step for producing secondary metabolites through plant cell culture (PCC) [13]. The various factors affect the biosynthesis of bioactive compounds in plants [7–10, 12, 14–17]. Amongst the available techniques for enhancing secondary metabolites biosynthesis in PCC, the elicitation is likely the most effective one for dramatic increment in yield [18, 19]. The mass biosynthesis of secondary metabolites even in plant cells overexpressed for key genes of biosynthetic pathway yet requires elicitation [20]. Among the various elicitors, fungal elicitors are widely applied for eliciting the biosynthesis of secondary metabolites in plant *in vitro* culture, as a result of their high effectuality and little toxicity on plant cells [21].

Endophytic fungi synthesize the conserved molecules known as microbe-associated molecular patterns (MAMPs). The first plant defense line is recognizing MAMPs by receptors on plant cell surface. Indeed, plant pattern recognition receptors localized on cell surface recognize MAMPs, and thus induce plant defense system [22–25]. The concentration levels and types of MAMPs are different in fungal cell extract (CE) and culture filtrate (CF). Accordingly, it seems that the potential of these fungal elicitors (CE and CF) is likely different in paclitaxel biosynthesis induction. The previous studies [8–10] demonstrated the positive influences of CE and CF of endophytic fungi on paclitaxel biosynthesis in cell suspension culture (CSC) of *C*. *avellana*. Nevertheless, no information is available regarding the simultaneous use of fungal CE and CF on secondary metabolites production in PCC including paclitaxel biosynthesis in *C*. *avellana* CSC. Fungal elicitor type, concentration and adding-time, and also exposure time of cell culture with it should be optimized to achieve the maximum biosynthesis of paclitaxel in *C*. *avellana* CSC [8–10]. However, the optimization of these factors is not only time-consuming but also costly.

Analyzing the relationship amongst input variables “CE and CF concentration levels, elicitor adding day and CSC harvesting time” and paclitaxel biosynthesis and secretion could help to optimize the conditions for the biosynthesis and secretion of this valuable secondary metabolite. Multivariate statistical approaches including stepwise regression (SR), ordinary least squares regression (OLSR), principal component regression (PCR) and partial least squares regression (PLSR) have been used in biological studies [26–29]. There are no studies to evaluate regression methods including SR, OLSR, PCR and PLSR to model and predict paclitaxel biosynthesis. SR is a well-known data-mining method selecting the explanatory variables for regression model from a group of input variables [30]. OLS is a statistical method estimating the relationship amongst independent variable(s) and dependent variable by minimizing sum of square differences among the predicted and observed values of dependent variable [31]. PCR is a regression method established on principal component analysis (PCA) [32]. PLSR, combining PCA and multiple regression, is a powerful modeling technique especially when the factors (input variables) are highly collinear [33]. Indeed, PLSR is an alternative for PCR which selects principal components that are related to independent variable [32].

The objectives of this research were (i) to evaluate the efficiency of CE and CF derived from endophytic fungus “*Camarosporomyces flavigenus*” isolated from *C*. *avellana*, either individually or as a combined treatment, on paclitaxel biosynthesis and secretion in *C*. *avellana* CSC, (ii) to estimate growth and paclitaxel biosynthesis (intracellular, extracellular and total) (model parameters) in *C*. *avellana* cell culture treated with fungal elicitors derived from *C*. *flavigenus* using regression methods (SR, OLS, PCR and PLSR) and (iii) to suggest the best regression model for prediction of growth, paclitaxel biosynthesis and its secretion in *C*. *avellana* cell culture.

## Material and methods

### Fungal and plant cell culture reagents

Culture medium components and paclitaxel standard applied in this research were purchased from Sigma (USA) and Merck (Germany) chemical companies.

### Cell suspension culture

Callus of *C*. *avellana* (ecotype Gerd Ashkorat) was produced from seed cotyledons on MS medium supplemented with 2 mg l^−1^ 2,4-Dichlorophenoxyacetic acid (2, 4-D) and 0.2 mg l^−1^ 6-Benzylaminopurine (BAP), and 8 g l^−1^ agar agar [7]. *C*. *avellana* CSC was established with cultivating 5 g fresh callus into 250 ml flasks containing 100 ml of the same medium and the cultures were maintained at 25°C in darkness on gyratory shakers at 110 rpm. CSCs were also subcultured until the cells reached homogeneity.

### Preparation of elicitors and elicitation experiment

Endophytic fungus applied in this research was a strain of *Camarosporomyces flavigenus*, HEF_17_, isolated from the leaf of *C*. *avellana* grown in Iran. CE and CF elicitors were prepared as described previously [8]. For elicitation, 1.5 ± 0.1 g of *C*. *avellana* cells (fresh mass) was cultured in 100 ml flasks containing 30 ml MS medium supplemented with 2 mg l^−1^ 2,4-D and 0.2 mg l^−1^ BAP.

Based on our previous studies [8–10], three concentrations (2.5, 5 and 10% (v/v)) of fungal elicitors “CE:CF (100:0, 75:25, 50:50, 25:75, 0:100 v/v)”, and also mid (day 13) and late (day 17) log phase of *C*. *avellana* cell cultures were selected for adding fungal elicitors. Control received an equal volume of water (for CE)/ potato dextrose broth (PDB) (for CF). Growth curve of *C*. *avellana* cells in the mentioned conditions has been given elsewhere [7].

### Cell growth measurement

Cell growth was determined by the measurement of cell dry weight (DW). Cell biomass was separated from culture medium by the filtration (Whatman No. 1) and washed with distilled water to remove the residual medium, afterward freeze-dried to constant weight by a vacuum-freeze drier.

### Quantification of paclitaxel

*C*. *avellana* cells were separated from culture medium by a filter paper (Whatman No. 1). Intracellular and extracellular paclitaxel were extracted from the cells and culture broth using a procedure described by Salehi et al. [7, 8, 12]. Filtering all samples was performed by 0.22 μm cellulose acetate syringe filters before HPLC analysis. Paclitaxel in the samples was analyzed by HPLC (Waters, USA) with a C18 analysis column (Machereye-Nagel EC 250/4.6 Nucleodur). Each sample (20 μl) was injected and detected at 230 nm using a UV detector. The mobile phase was methanol: water (80:20 v/v) at a flow rate of 1.0 ml/min. The quantification of paclitaxel was based on an external standard of genuine paclitaxel (Sigma).

### Experimental design

The experiment was planned based on randomized complete block design (RCBD) with factorial arrangement, three factors containing fungal elicitor type with 10 levels ((CE:CF (100:0, 75:25, 50:50, 25:75, 0:100 v/v) and water: PDB (100:0, 75:25, 50:50, 25:75, 0:100 v/v)”, elicitor concentration with three levels (2.5, 5, and 10% (v/v)), adding day with two levels (days 13 and 17), and three replicates. The cultures were harvested in 2-day intervals after elicitation until 23^rd^ day. It is noteworthy that fungal elicitors “100CE:0CF, 75CE:25CF, 50CE:50CF, 25CE:75CF and 0CE:100CF” were named as E^CE/CF^, i.e. E^100/0^, E^75/25^, E^50/50^, E^25/75^ and E^0/100^, respectively.

### Regression methods

The data were randomly divided into a training subset (70%) and testing one (30%), respectively. Training subset was applied to develop regression models, and testing subset was applied to test the predictability of developed models [34].

Stepwise regression (SR), ordinary least squares regression (OLSR), principal component regression (PCR) and partial least squares regression (PLSR) were used to predict DW, intracellular, extracellular and total yield of paclitaxel, and also extracellular paclitaxel portion. XLSTAT software [35] and Minitab [36] software were used for the development and evaluation of regression models. Also, the graphs were made by GraphPad Prism 5 [37] software.

### Model evaluation

The efficiency of regression models is assessed by five statistical criteria including value account for (VAF) (Eq (1)), root mean square error (RMSE) (Eq (2)), coefficient of determination (R^2^) (Eq (3)), mean absolute percentage error (MAPE) (Eq (4)) and relative percent difference (RPD) (Eq (5)).

$$VAF=\left[1-\frac{VAR\;(y_{act}-y_{est})}{VAR\;(y_{act})}\right]$$ (1)

$$RMSE=\sqrt{\left(\sum_{i=1}^{n}{\left(y_{est}-y_{act}\right)}^{2}\right)/n}$$ (2)

$$R^{2}=1-\left(\sum_{i=1}^{n}{\left(y_{est}-y_{act}\right)}^{2}/\sum_{i=1}^{n}{\left(y_{act}-\bar{y}\right)}^{2}\right)$$ (3)

$$MAPE=1/n\sum_{i=1}^{n}|\frac{\left(y_{act}-y_{est}\right)}{\left(y_{act}\right)}|\times 100$$ (4)

$$RPD=\frac{Standard\;deviation}{RMSE}$$ (5)

Where “y_act_” are the measured values, “y_est_” are the predicted values, and “n” is the number of data.

Evaluation of regression models was performed according to RPD values (Table 1) [38].

**Table 1:** Relative percent difference (RPD) values for evaluating models.

RPD	Model validation
**<1.0**	very poor models/predictions
**1.0 < RPD < 1.4**	poor models/predictions
**1.4 < RPD < 1.8**	fair models/predictions
**1.8 < RPD < 2.0**	good models/predictions
**2.0 < RPD < 2.5**	very good quantitative models/predictions
**RPD > 2.5**	excellent models/predictions

## Results and discussion

### Effects of elicitors derived from *C*. *flavigenus* on *C*. *avellana* cell growth

**Fig 1:**
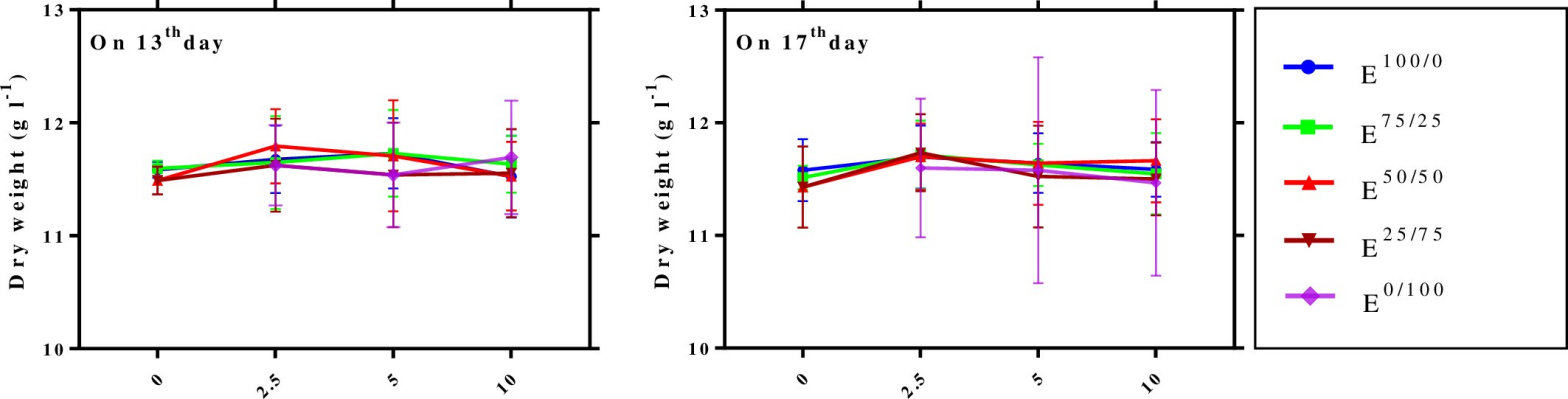
Effects of adding cell extract (CE) and/or culture filtrate (CF) derived from *Camarosporomyces flavigenus* on 13^th^ and 17^th^ days of culture cycle on cell growth of *Corylus avellana* L. average values are given, standard error are represented by vertical lines. E^100/0^; 100CE:0CF, E^75/25^; 75CE:25CF, E^50/50^; 50CE:50CF, E^25/75^; 25CE:75CF, E^0/100^; 0CE:100CF.

DW of the cells in *C*. *avellana* CSCs exposed with E^100/0^, E^75/25^, E^50/50^, E^25/75^ and E^0/100^ derived from *C*. *flavigenus* in mid (day 13) and late (day 17) log phase were measured as cell growth. Analysis of variance (ANOVA) demonstrated that the main effects of factors “elicitor type, concentration level and elicitor adding day” and also their reciprocal interactions on DW were not significant (Table 2), suggesting that fungal elicitors, regardless of its adding day and concentration levels, did not have an impact on cell growth in *C*. *avellana* CSC (Fig 1). It displays adaptability of *C*. *avellana* cells to elicitors derived from *C*. *flavigenus*, in view of the fact that strain HEF_17_ is a symbiont of this plant. It is reported that endophytic fungi display no negative effect on plant cell growth, and even a few of them are capable of enhancing the growth [8, 9, 39, 40].

**Table 2:** Analysis of variance for the effects of adding cell extract and culture filtrate of *Camarosporomyces flavigenus*, either individually or combined treatment, on 13^th^ and 17^th^ days of culture cycle on cell growth and paclitaxel biosynthesis in *Corylus avellana* L. cell culture.

Source of variation	Degree of freedom	Dry weight	Paclitaxel				
			Extracellular (μg l^-1^)	Intracellular (μg g^-1^ DW)	Intracellular (μg l^-1^)	Total (μg l^-1^)	Extracellular paclitaxel portion (%)
Block	2	0.120^ns^	238.527**	2.304*	448.667*	18254.390**	14.388*
Elicitor type (A)	9	0.088^ns^	3593.959**	89.035**	12029.703**	1322.076**	3.236^ns^
Concentration level (B)	2	0.157^ns^	5325.560**	120.652**	15693.842**	28725.210**	18.664*
Elicitor-adding time (C)	1	0.008^ns^	6448.534**	137.383**	18179.201**	39106.559**	18.484*
A × B	18	0.030^ns^	950.536**	18.466**	2460.363**	46282.223**	3.898^ns^
A × C	9	0.006^ns^	1029.614**	19.739**	2667.467**	6416.736**	2.977^ns^
B × C	2	0.001^ns^	1254.118**	17.680**	2339.284**	6964.934**	12.256 ^ns^
A × B × C	18	0.035^ns^	274.445**	3.170**	431.205**	6907.354**	2.123 ^ns^
Error	118	46.858	3109.738**	0.672	63.593	1359.712**	504.058

*, ** and ns indicate significant difference *p*<0.05, significant difference *p*<0.01 and non-significant, respectively.

### Effect of exposure time of *C*. *avellana* cells with fungal elicitor on paclitaxel biosynthesis

**Fig 2:**
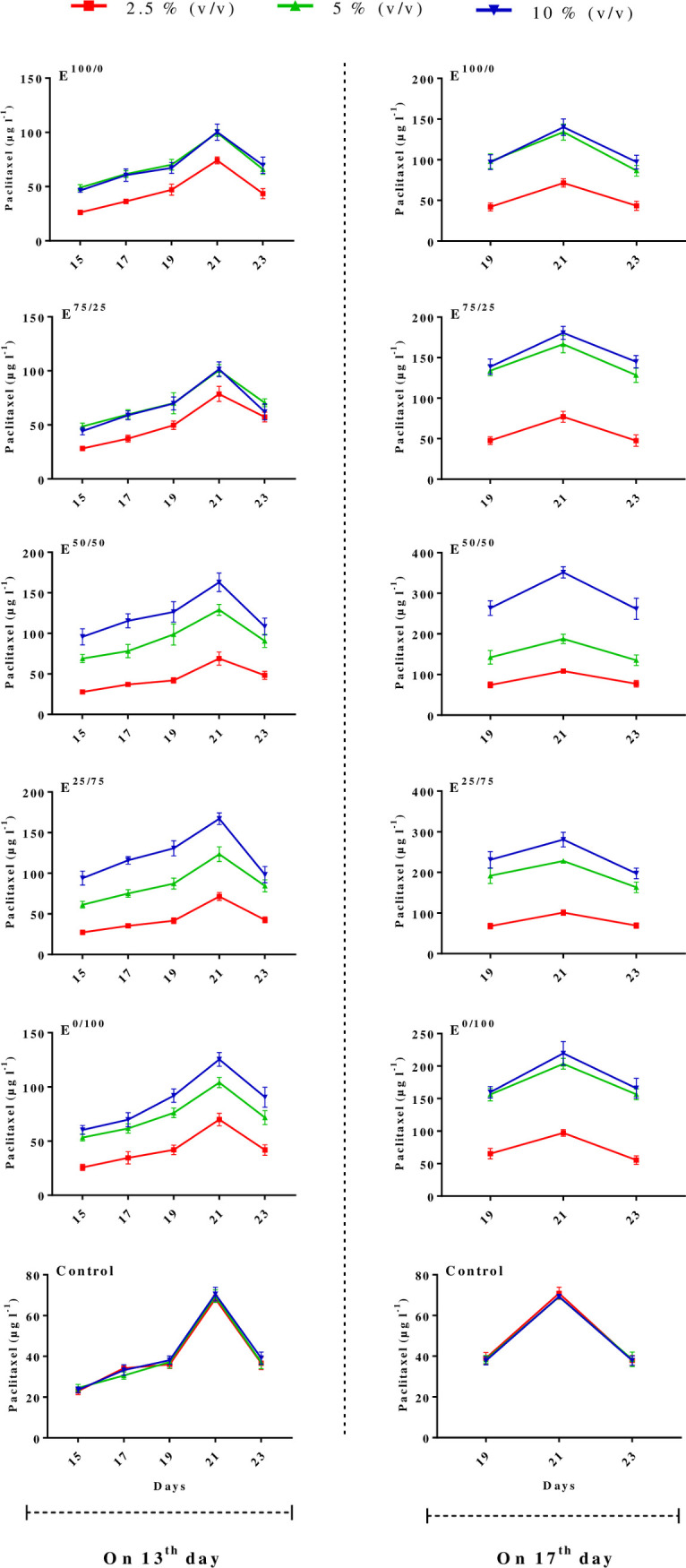
Time course of paclitaxel biosynthesis in *Corylus avellana* cell cultures exposed with 2.5, 5 and 10% (v/v) of cell extract (CE) and/or culture filtrate (CF) derived from *Camarosporomyces flavigenus* on 13^th^ and 17^th^ days of cell culture cycle. E^100/0^; 100CE:0CF, E^75/25^; 75CE:25CF, E^50/50^; 50CE:50CF, E^25/75^; 25CE:75CF, E^0/100^; 0CE:100CF.

To study the relations between paclitaxel biosynthesis and exposure time of *C*. *avellana* cells with E^100/0^, E^75/25^, E^50/50^, E^25/75^ and E^0/100^ derived from *C*. *flavigenus*, paclitaxel content in *C*. *avellana* CSCs treated with various concentration levels (2.5, 5 and 10% (v/v)) of five fungal elicitors at days 13 and 17 (mid and late log phase) were measured in 2-day periods after elicitation (Fig 2). Taken together, paclitaxel biosynthesis increment was recorded in the course of cell growth, and its highest statistically significant level was biosynthesized on day 21. Paclitaxel content in *C*. *avellana* CSCs reduced on 23^rd^ day, likely because of paclitaxel molecular structure changes or its degradation beginning. The degradation of bioactive molecules could happen either extra- or intracellularly [41]. Paclitaxel productivity (Pr) and paclitaxel biosynthesis elicitation index (ratio of elicited culture paclitaxel productivity to control one, Pr_e_/Pr_c_) at 2-day periods after elicitation showed that maximum paclitaxel biosynthesis elicitation in cultures treated with E^100/0^, E^75/25^, E^50/50^, E^25/75^ and E^0/100^ derived from *C*. *flavigenus* at days 13^th^ and 17^th^ was recorded at first days after treatment but later the effects of elicitors reduced (Fig 2).

### Effects of elicitors derived from *C*. *flavigenus* on paclitaxel biosynthesis

The effects of elicitors “E^100/0^, E^75/25^, E^50/50^, E^25/75^ and E^0/100^” derived from *C*. *flavigenus* on paclitaxel biosynthesis were surveyed in an elicitor adding time-, exposure time- and concentration level-dependent way. Maximum significant paclitaxel level was recorded on day 21 (Fig 2). Thus, this time point, day 21, was selected as the benchmark of paclitaxel biosynthesis. The results of paclitaxel biosynthesis induction in *C*. *avellana* cell cultures using elicitors derived from *C*. *flavigenus* displayed that the biosynthesis of this metabolite was significantly affected by above mentioned fungal elicitors (Table 2). The results of ANOVA showed that the main effects of factors (fungal elicitor type, concentration level and adding day) and their interactions (reciprocal and trilateral effects) on paclitaxel biosynthesis (intracellular, extracellular and total yield) were highly significant (*p* < 0.01) (Table 2). According to these significant interactions (Table 2), it can be concluded that fungal elicitor adding day and concentration levels affected paclitaxel biosynthesis variously at each elicitor type. Therefore, elicitor concentration level and its adding day were further scrutinized on each elicitor to carefully analyze these significant interactions.

### Effects of adding day and concentration level of fungal elicitors on paclitaxel biosynthesis

**Fig 3:**
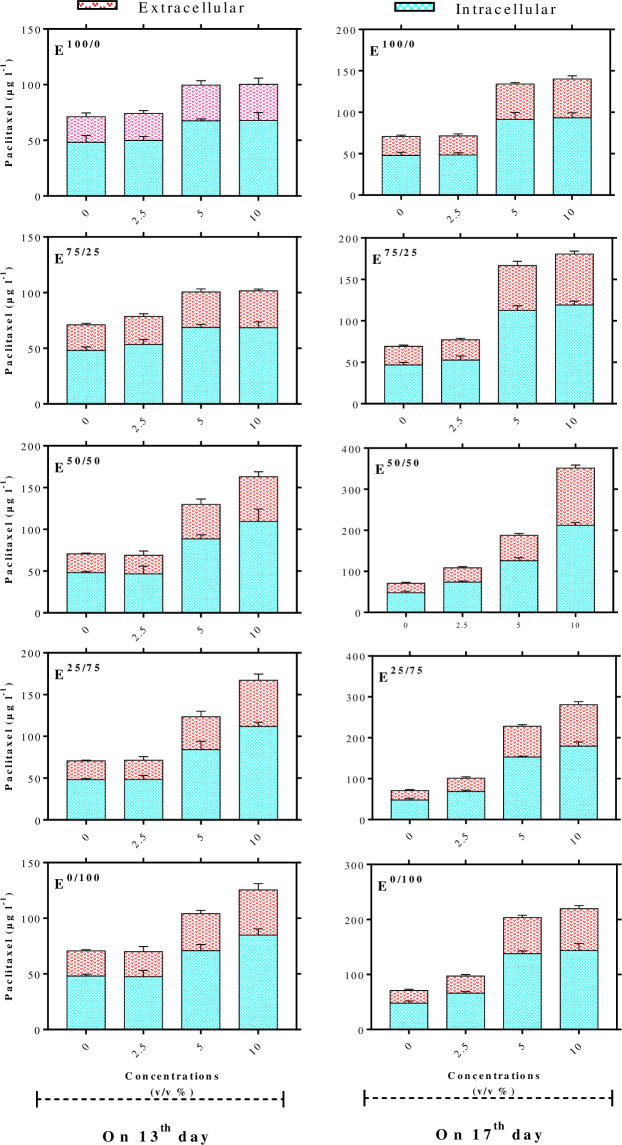
Effects of adding cell extract (CE) and/or culture filtrate (CF) derived from *Camarosporomyces flavigenus* at days 13 and 17 of culture cycle on paclitaxel biosynthesis in *Coryllus avellana* cell suspension culture. Average values are given, standard error are represented by vertical lines. E^100/0^; 100CE:0CF, E^75/25^; 75CE:25CF, E^50/50^; 50CE:50CF, E^25/75^; 25CE:75CF, E^0/100^; 0CE:100CF.

According to means comparison, adding 5 and 10% (v/v) E^100/0^ on 13^th^ day of cell culture cycle led to significantly higher paclitaxel biosynthesis (1.4-fold) as compared to control, but adding 2.5% (v/v) E^100/0^ on 13^th^ day did not significantly improve paclitaxel biosynthesis (Fig 3). In the same way, *C*. *avellana* cell cultures treated with 2.5% (v/v) E^100/0^ on 17^th^ day of cell culture cycle displayed no significant paclitaxel biosynthesis as compared to control (Fig 3). However, adding 5 and 10% (v/v) of this elicitor at day 17 significantly improved paclitaxel biosynthesis (137.04 μg l^-1^; 1.9-folds). Paclitaxel biosynthesis in *C*. *avellana* cell cultures treated with 5 and 10% (v/v) E^100/0^ in late log phase, day 17, were significantly higher (1.4-fold) than that in mid log phase. The optimized concentration level for E^100/0^ on 17^th^ day of cell culture cycle was 5% (v/v) (Fig 3).

As shown in Fig 3, adding 2.5% (v/v) E^75/25^ (1.875% (v/v) CE and 0.625% (v/v) CF) on 13^th^ and 17^th^ days did not affect paclitaxel biosynthesis in *C*. *avellana* CSC, whereas cell cultures exposed with 5 and 10% (v/v) of mentioned elicitor in mid and late log phase significantly enhanced paclitaxel biosynthesis. Adding 5 and 10% (v/v) E^75/25^ at day 17 resulted in the significantly higher contents of paclitaxel (1.7-fold) as compared to that at day 13. The most significant total yield of paclitaxel (166.6 μg l^−1^) in cell cultures subjected to E^75/25^ was biosynthesized using 5% (v/v) of this elicitor at day 17, about 2.3- fold that biosynthesized in control culture (Fig 3). It is noteworthy that no significant difference was observed between cell cultures treated with 5 and 10% (v/v) E^75/25^ at day 17.

The results displayed that cell cultures treated with 5 and 10% (v/v) E^50/50^ derived from *flavigenus* in mid log phase (day 13), and also 2.5, 5 and 10% (v/v) of it in late log phase (day 17) significantly increased paclitaxel biosynthesis in *C*. *avellana* cell CSCs. However, cell cultures exposed to 10% (v/v) of this elicitor (5% (v/v) CE and 5% (v/v) CF) on 17^th^ day displayed a pronounced increment in paclitaxel content (4.8-fold) than control, measured 351.4 μg l^−1^. Cell cultures exposed with 2.5, 5 and 10% (v/v) E^50/50^ at day 17 displayed significantly higher paclitaxel biosynthesis (1.6, 1.4 and 2.2, respectively) than that at day 13 (Fig 3).

As illustrated in Fig 3, adding different concentration levels of E^25/75^ derived from *C*. *flavigenus* in mid and late log phase (except 2.5% (v/v) of it at day 13) resulted in the higher significantly biosynthesis of paclitaxel as compared to control culture. Paclitaxel biosynthesis in cell cultures treated with 5 and 10% (v/v) E^25/75^ derived from *C*. *flavigenus* in late log phase (day 17) were significantly higher (1.8- and 1.7-fold, respectively) than that in mid log phase (day 13) (Fig 3).

According to mean comparison, *C*. *avellana* cell cultures treated with 5 and 10% (v/v) E^0/100^ in mid log phase (day 13), and also 2.5, 5 and 10% (v/v) of it in late log phase (day 17) displayed a significant increment in paclitaxel biosynthesis (Fig 3). As Fig 3 shows adding 2.5, 5 and 10% (v/v) E^0/100^ at day 17 resulted in the significantly higher contents of paclitaxel (1.4-, 2.0- and 1.8-fold, respectively) than that on day 13. The maximum significant total yield of paclitaxel in cell cultures exposed to E^0/100^ (203.5 μg l^−1^) was biosynthesized using 5% (v/v) of it on day 17, about 2.8-fold that biosynthesized in control culture (Fig 3).

The maximum total content of paclitaxel in *C*. *avellana* cell cultures treated with three concentration levels (2.5, 5 and 10% (v/v) of five fungal elicitors derived from *C*. *flavigenus* E^100/0^, E^75/25^, E^50/50^, E^25/75^ and E^0/100^ (351.4 μg l^−1^) was biosynthesized using 10% (v/v) E^50/50^ containing 5% (v/v) CE and 5% (v/v) CF on 17^th^ day of cell culture cycle, about 4.8- fold that produced in control culture (Fig 3). Extracellular and intracellular paclitaxel of cell culture treated with 10% (v/v) E^50/50^ on day 21 were 212.3 μg L^−1^ (4.2-fold) and 139.1 (6.0-fold), respectively (Fig 3). By comparison, 10% (v/v) of individual treatment of CE (E^100/0^) and CF (E^0/100^) elicited paclitaxel biosynthesis only 1.9- and 3.0-fold higher than control, respectively, while 10% (v/v) of their combined treatment “E^50/50^” induced paclitaxel biosynthesis 4.8-fold higher than control (Fig 3). The different types and high concentrations of MAMPs in E^50/50^ than E^100/0^ and E^0/100^ likely resulted in higher paclitaxel biosynthesis in *C*. *avellana* CSC treated with whole fungal elicitors as compared to that with individual elicitors.

In this research, whole fungal elicitors (the combined treatment of fungal CE and CF) have been used for the elicitation of secondary metabolite biosynthesis in PCC for the first time. The results clearly showed the high potential of whole fungal elicitors for paclitaxel biosynthesis increment in *C*. *avellana* cell culture. Various fungal elicitors applied in this research led to the different responses regarding paclitaxel biosynthesis elicitation. E^50/50^ consisting 5% (v/v) CE and 5% (v/v) CF derived from *C*. *flavigenus* strain HEF_17_, isolated from *C*. *avellana* leaf, was an efficient elicitor for biosynthesizing paclitaxel in *C*. *avellana* cell culture. Endophytic fungi synthesize conserved molecules known as microbe-associated molecular patterns (MAMPs) that can activate plant defense response [22, 42]. Indeed, the recognition of MAMPs via plant cell surface receptors is the initial phase of defense response induction. The particular structure of receptors leads to particularly recognize specific MAMPs [43]. The specific and diverse responses of plant cells to different fungal elicitors in the elicitation of secondary metabolite biosynthesis as seen in our research can be due to unique interactions of plant cell-surface receptors with fungal MAMPs [44].

**Fig 4:**
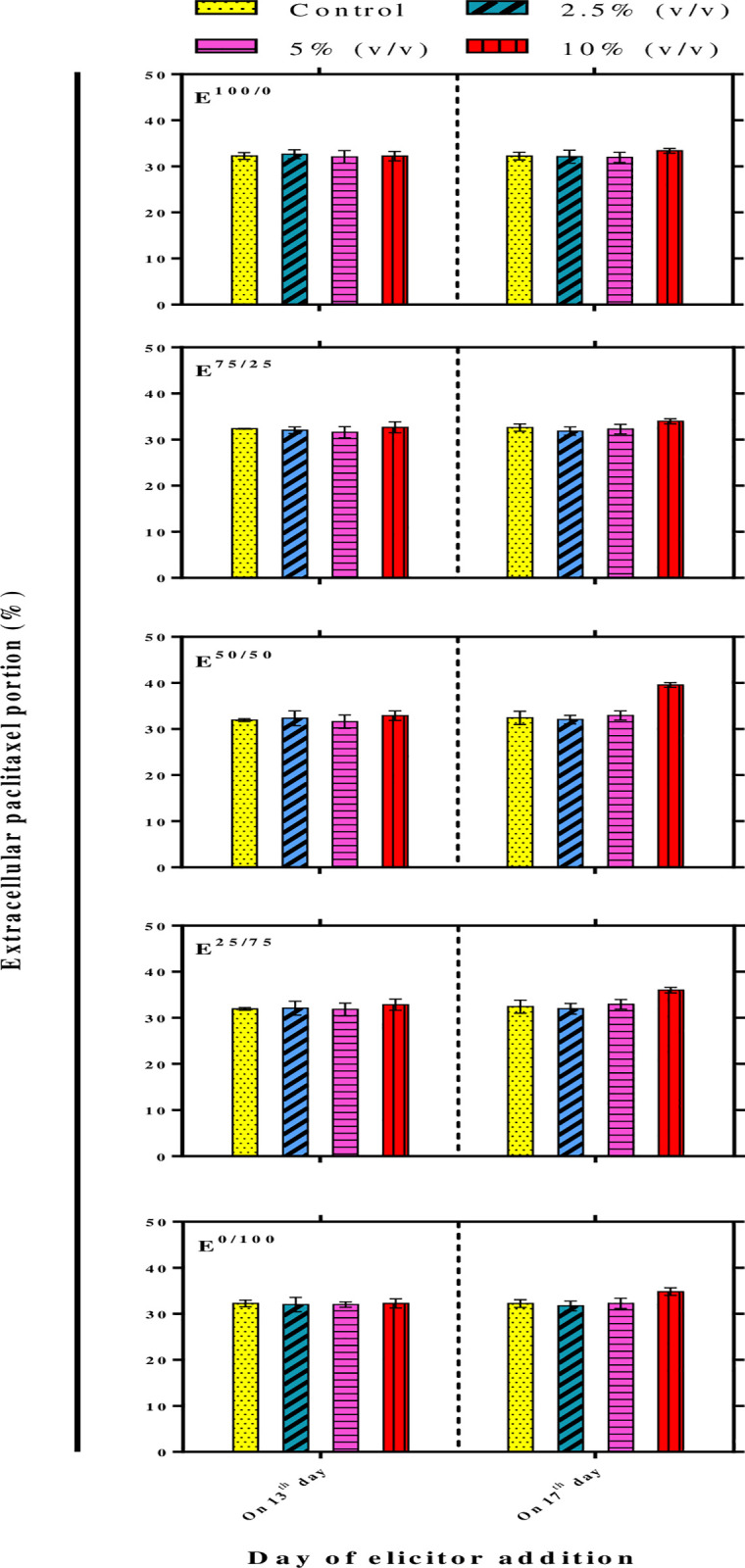
Extracellular paclitaxel portion (%) in *Corylus avellana* cell suspension culture exposed with 2.5, 5 and 10% (v/v) of cell extract (CE) and/or culture filtrate (CF) derived from *Camarosporomyces flavigenus*on on 13^th^ and 17^th^ days of cell culture cycle. E^100/0^; 100CE:0CF, E^75/25^; 75CE:25CF, E^50/50^; 50CE:50CF, E^25/75^; 25CE:75CF, E^0/100^; 0CE:100CF.

Our results illustrated that paclitaxel secretion was also increased by E^50/50^, 24.1% more than that in control (Fig 4). Paclitaxel secretion from the cells to culture medium decreases toxicity and feedback inhibition of paclitaxel [10, 45]. Besides, the secretion of paclitaxel to culture medium undoubtedly makes easy extraction and the purification of it which is required for the steady production of paclitaxel at the commercial level. The influence of fungal metabolites on plant cell membrane transporters likely leads to secrete paclitaxel from the cells into culture medium that is a key strategy for a continuous production system.

The various fungal elicitors have been used in *C*. *avellana* cell culture to enhance paclitaxel productivity. *C*. *avellana* cell culture treated with CF (2.5% (v/v) on 17^th^ day) of *Paraconiothyrium brasiliense* strain HEF_114_ isolated from *C*. *avellana* displayed a 3.0-folds increment in paclitaxel biosynthesis [8]. Also, *C*. *avellana* cell culture subjected to CE (10% (v/v) on 17^th^ day) of *Chaetomium globosum* strain YEF_20_ led to a 4.1-fold increment in paclitaxel biosynthesis [8]. In another report, the addition of 2.5% (v/v) CE of *Epicoccum nigrum* strain YEF_2_ to *C*. *avellana* CSC resulted in a 3.6-fold in paclitaxel biosynthesis [9]. In another attempt to find the efficient elicitors, out of different fungal elicitors, 5% (v/v) CE and also 2.5 and 5% (v/v) of cell wall (CW) derived from *Coniothyrium palmarum* added at day 17 resulted in maximum paclitaxel biosynthesis (3.6-folds), while a combined treatment of CW (2.5% (v/v) on 17^th^ day) and 50 mM of methyl-β–cyclodextrin (MBCD) highly enhanced paclitaxel biosynthesis (5.8-fold) in *C*. *avellana* cell culture with displaying a synergistic effect [10]. In another report, a slight increase in paclitaxel biosynthesis displayed in *C*. *avellana* cell culture affected by silver nanoparticles [46]. Also, the joint effects of phenylalanine (3 μM) and vanadyl sulfate (0.05 and 0.1 mM) in culture medium supplemented with fructose (3% (v/v)) led to a 2.3-fold increment in paclitaxel biosynthesis [47].

In the light of the prominent positive effect of MBCD on paclitaxel biosynthesis, it can be suggested to assess the effects of whole fungal elicitors (E^50/50^ consisting 5% (v/v) CE and 5% (v/v) CF) and MBCD, in a combined treatment on paclitaxel productivity in *C*. *avellana* cell culture.

Regardless of previous studies on the effects of fungal CE and CF on paclitaxel biosynthesis and secretion in *C*. *avellana* CSC, there was still the question that needed to be answered; whether whole fungal elicitors (the combined treatment of CE and CF) would lead to a higher enhancement of paclitaxel biosynthesis as compared to individual fungal elicitor? This research introduced “whole fungal elicitors” as an efficient treatment for boosting the biosynthesis of secondary metabolites in plant *in vitro* culture, paclitaxel biosynthesis in *C*. *avellana* CSC as a case study.

Finally, it is important to note that paclitaxel biosynthesis in *C*. *avellana* cell culture was not as high as that reported for *Taxus*. However, fast-growing *in vitro* culture of *C*. *avellana* and a series of genetic manipulation may compensate for the lower yield of paclitaxel in *C*. *avellana*.

### Regression models

All regression models (SR, OLSR, PCR and PLSR) displayed statistically significant relationships between each output variables (DW, intracellular paclitaxel, extracellular paclitaxel, total yield of paclitaxel and extracellular paclitaxel portion) and input variables (CE and CF concentration levels, adding day and harvesting day) (Table 3). SR, OLSR, PCR and PLSR models developed for output variables “DW, intracellular paclitaxel, extracellular paclitaxel, total yield of paclitaxel and extracellular paclitaxel portion” regarding CE and CF concentration levels, elicitor adding day and CSC harvesting day were shown in Table 3. Goodness-of-fit test of SR, OLSR, PCR and PLSR models was performed to detect the best model for predicting each output variables (DW, intracellular paclitaxel, extracellular paclitaxel, total yield of paclitaxel and extracellular paclitaxel portion). High VAF, R^2^ and RPD values and low RMSE and MAPE values displayed the model capability.

**Table 3:** Stepwise regression (SR), ordinary least squares regression (OLSR), principal component regression (PCR) and partial least squares regression (PLSR) for modeling growth (dry weight), paclitaxel biosynthesis and its secretion in *Corylus avellana* cell cultures (CSC) treated with fungal elicitors via cell extract (CE) and culture filtrate (CF) concentration levels (% (v/v)), elicitor adding day (AD) and CSC harvesting day (HD) of cell culture.

Measured parameters	Regression model	VAF	RMSE	R^2^	MAPE	RPD	Pr > F
**Dry weight (g l**^**-1**^**)**	SR	0.648	1.004	0.647	8.624	1.678	< 0.0001
	OLSR	0.670	0.967	0.670	8.225	1.729	< 0.0001
	PCR	0.670	0.967	0.670	8.225	1.729	< 0.0001
	PLSR	0.670	0.958	0.670	8.225	1.745	< 0.0001
	SR	-0.795 + 0.104X_AD_ + 0.476X_HD_					
	OLSR	-0.818–0.0002*X_CE_− 0.019X_CF_ + 0.100X_AD_+ 0.485X_HD_					
	PCR	-0.818–0.0002*CE– 0.019X_CF_ + 0.100X_AD_ + 0.485X_HD_					
	PLSR	-0.818–0.0002*CE—0.019X_CF_ + 0.100X_AD_ + 0.485X_HD_					
**Intracellular paclitaxel (**μ**g gDW**^**-1**^**)**	SR	0.622	2.396	0.623	32.749	1.616	< 0.0001
	OLSR	0.572	2.336	0.573	32.300	1.518	< 0.0001
	PCR	0.572	2.336	0.573	32.300	1.518	< 0.0001
	PLSR	0.572	2.314	0.572	32.300	1.532	< 0.0001
	SR	-5.428 + 0.688X_CE_ + 0.662X_CF_ + 0.942X_AD_ - 0.260X_HD_					
	OLSR	-4.768 + 0.439X_CE_ + 0.794X_CF_ + 0.828X_AD_ - 0.217X_HD_					
	PCR	-4.768 + 0.439X_CE_ + 0.794X_CF_ + 0.828X_AD_ - 0.217X_HD_					
	PLSR	-4.768 + 0.439X_CE_ + 0.794X_CF_ + 0.828X_AD_ - 0.217X_HD_					
**Extracellular paclitaxel (**μ**g l**^**-1**^**)**	SR	0.678	16.105	0.678	49.941	1.749	< 0.0001
	OLSR	0.620	15.821	0.620	47.578	1.611	< 0.0001
	PCR	0.620	15.821	0.620	47.578	1.611	< 0.0001
	PLSR	0.620	15.672	0.620	47.578	1.626	< 0.0001
	SR	-134.829 + 4.375X_CE_ + 3.929X_CF_ + 6.832X_AD_ +2.448X_HD_					
	OLSR	-123.859 + 2.679X_CE_ + 4.748X_CF_ + 5.847X_AD_ + 2.557X_HD_					
	PCR	-123.859 + 2.679X_CE_ + 4.748X_CF_ + 5.847X_AD_ + 2.557X_HD_					
	PLSR	-123.859 + 2.679X_CE_ + 4.748X_CF_ + 5.847X_AD_ +2.557X_HD_					
**Total yield of paclitaxel (**μ**g l**^**-1**^**)**	SR	0.650	41.796	0.650	36.372	1.676	< 0.0001
	OLSR	0.596	40.670	0.596	35.540	1.561	< 0.0001
	PCR	0.596	40.670	0.596	35.540	1.561	< 0.0001
	PLSR	0.596	40.289	0.596	35.540	1.575	< 0.0001
	SR	-263.513 + 11.32X_CE_ + 10.636X_CF_ + 17.827X_AD_ + 2.507X_HD_					
	OLSR	-242.508 + 7.120X_CE_ + 12.744X_CF_ + 15.505X_AD_ + 3.022X_HD_					
	PCR	-242.508 + 7.120X_CE_ + 12.744X_CF_ + 15.505X_AD_ + 3.022X_HD_					
	PLSR	-242.508 + 7.120X_CE_ + 12.744X_CF_ + 15.505X_AD_ + 3.022X_HD_					
**Extracellular paclitaxel portion (**μ**g l**^**-1**^**)**	SR	0.858	2.607	0.858	6.614	2.632	< 0.0001
	OLSR	0.860	2.513	0.860	6.390	2.653	< 0.0001
	PCR	0.860	2.513	0.860	6.390	2.653	< 0.0001
	PLSR	0.860	2.490	0.860	6.390	2.679	< 0.0001
	SR	-19.487 + 0.348X_CE_ + 0.256X_CF_ + 0.395X_AD_ + 2.211X_HD_					
	OLSR	-18.689 + 0.199X_CE_ + 0.319X_CF_ + 0.313X_AD_ + 2.223X_HD_					
	PCR	-18.689 + 0.199X_CE_ + 0.319X_CF_ + 0.313X_AD_ + 2.223X_HD_					
	PLSR	-18.689 + 0.199X_CE_ + 0.319X_CF_ + 0.313X_AD_ + 2.223X_HD_					

VAF; value account for, RMSE; root mean square error, R^2^; coefficient of determination, MAPE; mean absolute percentage error, RPD; relative percent difference

As shown in Table 3, SR had slightly higher VAF, R^2^ and RPD and lower RMSE and MAPE values as compared to OLSR, PCR and PLSR for predicting paclitaxel biosynthesis (intracellular, extracellular and total) and secretion. However, OLSR, PCR and PLSR displayed slightly higher VAF, R^2^ and RPD and lower RMSE and MAPE values as compared to SR. Goodness-of-fit displayed no difference regarding the accuracy of OLSR, PCR and PLSR for all output variables, 0.67, 0.57, 0.62, 0.60 and 0.86 for DW, intracellular paclitaxel, extracellular paclitaxel, total yield of paclitaxel and extracellular paclitaxel portion, respectively for training subset (Table 3). R^2^ values for predicting DW, intracellular paclitaxel, extracellular paclitaxel, total yield of paclitaxel and extracellular paclitaxel portion using SR models were estimated 0.65, 0.62, 0.68, 0.65 and 0.85, respectively for training subset (Table 3).

**Fig 5:**
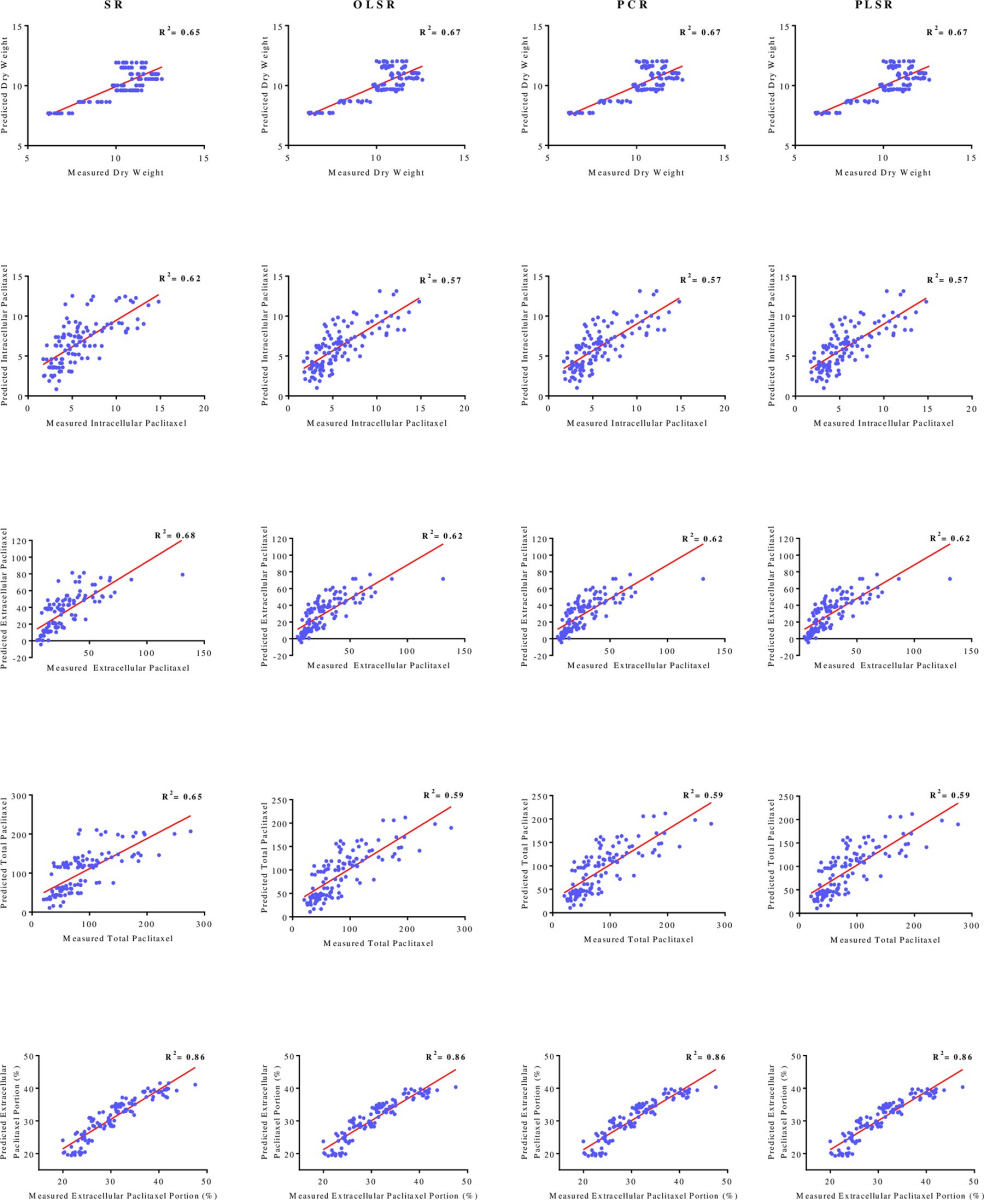
Scatter plot of measured data against predicted values of dry weight, intracellular, extracellular and total yield of paclitaxel, and extracellular paclitaxel portion in *Corylus avellana* cell cultures using stepwise regression (SR), ordinary least squares regression (OLSR), principal component regression (PCR) and partial least squares regression (PLSR) models in testing subset. The solid line shows fitted simple regression line on scatter points.

The fit of regression models was presented by R^2^ (Fig 5) for testing subset, suggesting the best-mentioned models can explain 67, 62, 68, 65 and 86% of the variability in intracellular paclitaxel, extracellular paclitaxel, total yield of paclitaxel and paclitaxel extracellular portion, respectively, when they faced unseen data.

In this study, multiple regression models were applied to determine the relationships among four factors “CE and CF concentration levels, elicitor adding day and CSC harvesting time” and each of parameters “DW, intracellular, extracellular and total yield of paclitaxel and extracellular paclitaxel portion”, and also the possibility of predicting of paclitaxel biosynthesis by the determining factors. Such mathematical predictions have been applied to predict cell growth and paclitaxel biosynthesis in *C*. *avellana* cell culture for the first time.

According to RPD, all developed regression (SR, OLSR, PCR and PLSR) displayed superior ability for predicting extracellular paclitaxel portion (Tables 1 and 3). Based on VAF, RMSE, R^2^, MAPE and RPD (Table 3), it can be concluded that mentioned regression models effectively worked only for modeling and predicting extracellular paclitaxel portion in *C*. *avellana* cell culture.

Since mentioned various regression methods showed fair predictive and fitting ability for growth and paclitaxel biosynthesis (Tables 2 and 3). It is recommended that artificial intelligence is used for accurate modeling and predicting growth and paclitaxel biosynthesis.

## Conclusion

This research presents the high potential of whole fungal elicitors (the combined treatment of CE and CF) derived from *C*. *flaveginus* strain HEF_17_ isolated from *C*. *avellana* for paclitaxel biosynthesis induction in *C*. *avellana* cell culture. The combined treatment of fungal CE and CF led to more paclitaxel biosynthesis than the individual use of them, suggesting the presence of different MAMPs with different concentrations in fungal CE and CF. High RPD shows the superior ability of regression models for predicting extracellular paclitaxel portion. Great accordance between the predicted and observed values of extracellular paclitaxel portion (Fig 5) supports the excellent performance of developed SR, OLSR, PCR and PLSR models for predicting extracellular paclitaxel portion.
